# Cytogenetic Response to Asciminib in Chronic Myeloid Leukemia With the e19a2 Micro BCR::ABL1 Transcript: A Case Report

**DOI:** 10.7759/cureus.83363

**Published:** 2025-05-02

**Authors:** Takuya Terakawa, Yasuhiro Shingai, Yoshiki Matsuoka, Yuka Amemiya, Wataru Nakahara, Yuma Tada, Sayako Yuda, Shigeo Fuji, Jun Ishikawa, Takafumi Yokota

**Affiliations:** 1 Hematology, Osaka International Cancer Institute, Osaka, JPN; 2 Oncology, Graduate School of Medicine, Osaka University, Suita, JPN

**Keywords:** asciminib, chronic myeloid leukemia, complete cytogenetic response, e19a2, micro bcr::abl1 transcript

## Abstract

Chronic myeloid leukemia (CML) is classified into three subtypes based on the *BCR* breakpoint, the rarest of which is micro *BCR::ABL1* (also known as e19a2 *BCR::ABL1*), which encodes a P230 fusion protein. CML patients with the e19a2 transcript are known to have a poor prognosis. Although second-generation tyrosine kinase inhibitors (TKIs) may be effective for this subtype, asciminib, a novel *BCR::ABL1* inhibitor that specifically targets the ABL1 myristoyl pocket, has only been shown to be effective in patients with the major *BCR::ABL1* transcript, with limited data on the micro *BCR::ABL1 *subtype. Here, we report a case of a CML patient with the e19a2 transcript who was intolerant to four TKIs but achieved complete cytogenetic response with asciminib. Our case suggests that asciminib, in addition to showing promising outcomes in CML patients with the major *BCR::ABL1* transcript, is an effective treatment option for CML patients with the e19a2 micro *BCR::ABL1* transcript.

## Introduction

Chronic myeloid leukemia (CML) is classified as a myeloproliferative neoplasm according to the World Health Organization (WHO) classification [1]. It is characterized by the presence of the Philadelphia chromosome, which results from a translocation between the *BCR* gene on chromosome 22 and the *ABL1* gene on chromosome 9 [2]. The *BCR::ABL1* fusion gene is classified into three subtypes based on its breakpoint on the *BCR* gene. Those with breakpoints in exons 12-16 are classified as major *BCR::ABL1* (most cases are exon 14 or 13 and are designated e14a2 or e13a2), exons 1-2 as minor *BCR::ABL1* (most cases are exon 1 and are designated e1a2), and exons 17-20 as micro *BCR::ABL1* (most cases are exon 19 and are designated e19a2). The fusion proteins translated from these mRNA transcripts are designated P210, P190, and P230, respectively [2,3]. The micro *BCR::ABL1* type of CML is the rarest subtype, accounting for approximately 0.3%-0.4% of all CML cases [4,5]. CML with the micro *BCR::ABL1* transcript was first described in 1990 by Saglio et al. [6]. Early studies showed that its clinical features were characterized by mild leukocytosis, a high platelet count, no or mild splenomegaly, low incidence of blasts in the peripheral blood, less frequent progression to the blast phase, and a generally better prognosis than typical CML [7,8]. However, recent reports have shown lower response rates to imatinib, as well as a poorer prognosis compared to the major *BCR::ABL1* type of CML [5,9,10].

In patients with major-type CML, quantitative polymerase chain reaction (PCR) for the major *BCR::ABL1* transcript, standardized to the International Scale (IS), is the recommended method for monitoring treatment response [11,12]. Although tyrosine kinase inhibitors (TKIs) have improved clinical outcomes, resistance and intolerance remain major challenges. Asciminib is a novel therapeutic agent for CML, an inhibitor that specifically targets the ABL1 myristoyl pocket (or STAMP inhibitor) and has shown superior efficacy and safety compared to bosutinib in patients who are resistant or intolerant to two or more TKIs [13,14]. The US Food and Drug Administration (FDA) approved asciminib in 2021 for this patient population. In addition, asciminib has demonstrated superior outcomes compared to conventional TKIs in patients with newly diagnosed CML [15]. Studies demonstrating the efficacy of asciminib have focused on CML patients with the major *BCR::ABL1* transcript. Its effect on other CML subtypes remains unknown because those subtypes are rare, making large-scale studies difficult to conduct. Given the higher resistance rates in relation to imatinib and potentially poor treatment outcomes in CML patients with the e19a2 micro *BCR::ABL1* transcript compared with typical CML [5,9,10], reports on the efficacy of asciminib in this rare subtype are important. In our case, a CML patient with the e19a2 micro *BCR::ABL1* transcript who was intolerant to four TKIs achieved complete cytogenetic response (CCyR) with asciminib. In this context, CCyR is defined as the absence of the Philadelphia chromosome in at least 20 metaphase bone marrow cells, or fewer than 1% *BCR::ABL1*-positive nuclei by interphase fluorescence in situ hybridization (FISH) on peripheral blood analysis [16].

## Case presentation

In May 2009, a Japanese male patient in his 70s whose medical history included postoperative prostate cancer, postoperative gastric cancer, immune thrombocytopenia, and angina pectoris was diagnosed with chronic-phase CML. His hemoglobin level was 13.7 g/dL; his white blood cell count was 23.6 × 10^9^/L (blasts: 0%; promyelocytes: 0%; myelocytes: 5.5%; band-form neutrophils: 3.0%; segmented neutrophils: 57.5%; monocytes: 1.0%; basophils: 15.5%; eosinophils: 6.0%; lymphocytes: 11.5%), and his platelet count was 552 × 10^9^/L (Table 1). He had no hepatosplenomegaly, and in his bone marrow cells, the blast count was 2.6%. All 20 mitotic cells analyzed were 45, X, -Y, t(9;22)(q34;q11.2). Interphase fluorescence in situ hybridization (FISH) on peripheral blood showed 82% *BCR::ABL1*-positive cells; however, reverse transcription polymerase chain reaction (RT-PCR) was negative for either major *BCR::ABL1 *or minor *BCR::ABL1* mRNA transcript, suggesting micro *BCR::ABL1* type CML. At that time, routine clinical RT-PCR for the e19a2 micro *BCR::ABL1* mRNA transcript was unavailable in Japan. He was treated with imatinib 400 mg daily for chronic-phase CML. He achieved CCyR but the dosage of imatinib was reduced to 300 mg daily due to thrombocytopenia, in January 2011. In October 2012, FISH on peripheral blood showed 19% *BCR::ABL1*-positive cells; the treatment was switched from imatinib to nilotinib 300 mg twice daily, and he reached CCyR. However, nilotinib treatment was discontinued in June 2015 after the onset of right internal carotid artery stenosis, cerebral infarction, and atrial fibrillation. Bosutinib 100 mg daily was started in March 2016 but was discontinued in April 2016 due to hepatotoxicity, followed by dasatinib 20 mg daily, which was started in June 2016. One month later, dasatinib was discontinued due to a transient ischemic attack. Bosutinib was reintroduced in September 2016 and the dosage was gradually increased from 100 mg up to 300 mg and CCyR was reached; however, bosutinib was discontinued in October 2022 because of acute heart failure caused by myocardial ischemia.

**Table 1:** Laboratory findings at the time of diagnosis and starting asciminib PT: prothrombin time; APTT: activated partial thromboplastin time; N/A: not available

Parameter	May 2009 (at the time of diagnosis)	October 2023 (at the time of starting asciminib)	Reference range
Biochemistry			
Albumin	4.5 g/dL	4.6 g/dL	4.1-5.1 g/dL
Aspartate transaminase	26 U/L	16 U/L	13-30 U/L
Alanine transaminase	17 U/L	9 U/L	10-42 U/L
Lactate dehydrogenase	376 U/L	252 U/L	124-222 U/L
Uric acid	6.9 mg/dL	4.0 mg/dL	3.7-7.8 mg/dL
Total bilirubin	0.50 mg/dL	0.40 mg/dL	0.4-1.5 mg/dL
Creatinine	0.89 mg/dL	1.21 mg/dL	0.65-1.07 mg/dL
Blood urea nitrogen	18 mg/dL	24 mg/dL	8-20 mg/dL
Sodium	141 mmol/L	141 mmol/L	138-145 mmol/L
Potassium	4.5 mmol/L	4.2 mmol/L	3.6-4.8 mmol/L
Chloride	104 mmol/L	102 mmol/L	101-108 mmol/L
Glucose	86 mg/dL	109 mg/dL	73-109 mg/dL
Hemoglobin A1c	N/A	6.7%	4.9-6.0%
Total cholesterol	154 mg/dL	173 mg/dL	142-248 mg/dL
Triglyceride	168 mg/dL	282 mg/dL	40-234 mg/dL
C-reactive protein	0.04 mg/dL	0.05 mg/dL	0-0.14 mg/dL
Complete blood count			
White blood cells	23.6 x 10^9^/L	6.0 x 10^9^/L	3.3-8.6 x 10^9^/L
Red blood cells	5.13 x 10^12^/L	4.39 x 10^12^/L	4.35-5.55 x 10^12^/L
Hemoglobin	13.7 g/dL	14.3 g/dL	13.7-16.8 g/dL
Hematocrit	43.1%	44.1%	40.7-50.1%
Platelet	552 x 10^9^/L	65 x 10^9^/L	158-348 x 10^9^/L
Differential count			
Blast cells	0%	0%	0-0.1%
Promyelocytes	0%	0%	0.-0.1%
Myelocytes	5.5%	2.5%	0-0.4%
Band neutrophils	3.0%	1.0%	0.5-6.5%
Segmented neutrophils	57.5%	76.5%	38-74%
Monocytes	1.0%	3.0%	2-10%
Eosinophils	6.0%	4.0%	0-8.5%
Basophils	15.5%	1.5%	0-2.5%
Lymphocytes	11.5%	11.5%	16.5-49.5%
Coagulation			
PT	82%	115%	70-130%
APTT	32.6 sec	28.1 sec	24-39 sec
D-dimer	<0.1 μg /mL	0.4 μg /mL	0-0.9 μg /mL

**Figure 1:**
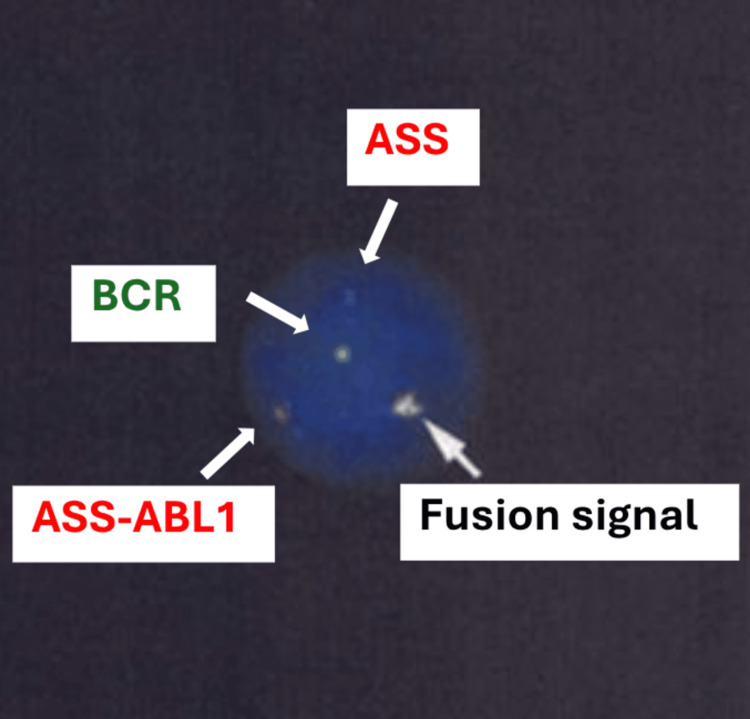
Fluorescence in situ hybridization (FISH) analysis for BCR::ABL1 FISH analysis of peripheral blood showing *argininosuccinate synthetase *(*ASS*)-*ABL1 *(red signals), *BCR* (green signals), and *BCR-ABL1* fusion signals (yellow) in interphase nuclei.

**Figure 2:**
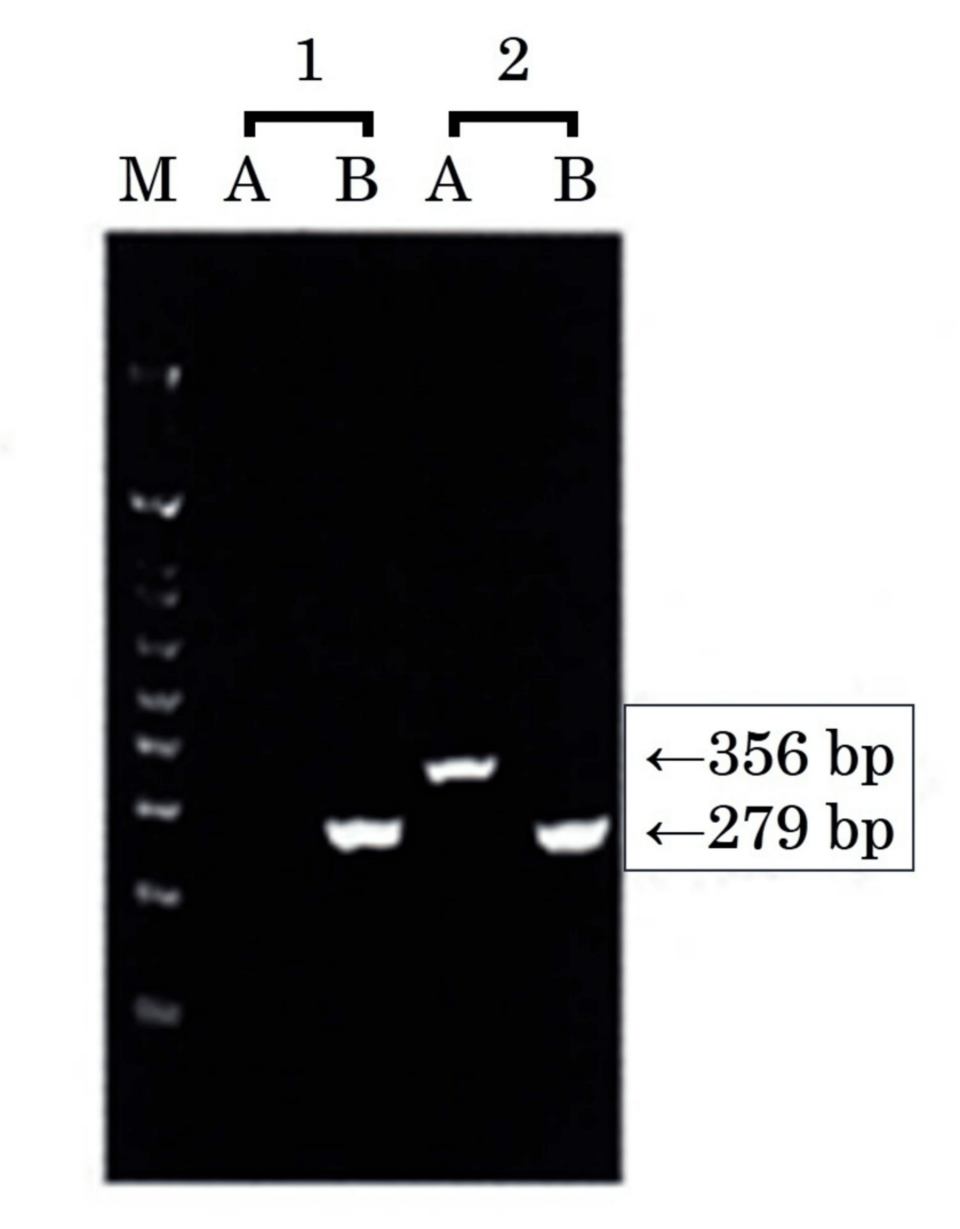
Qualitative reverse transcription polymerase chain reaction for the e19a2 micro BCR::ABL1 transcript Qualitative reverse transcription polymerase chain reaction testing showed that the e19a2 micro *BCR::ABL1* transcript was detected. M: size marker (1 kb plus DNA ladder); 1: negative control; 2: sample. Amplification band sizes: A: e19a2 micro *BCR::ABL1* mRNA 356 bp; B: *beta-actin* mRNA 279 bp.

**Figure 3:**
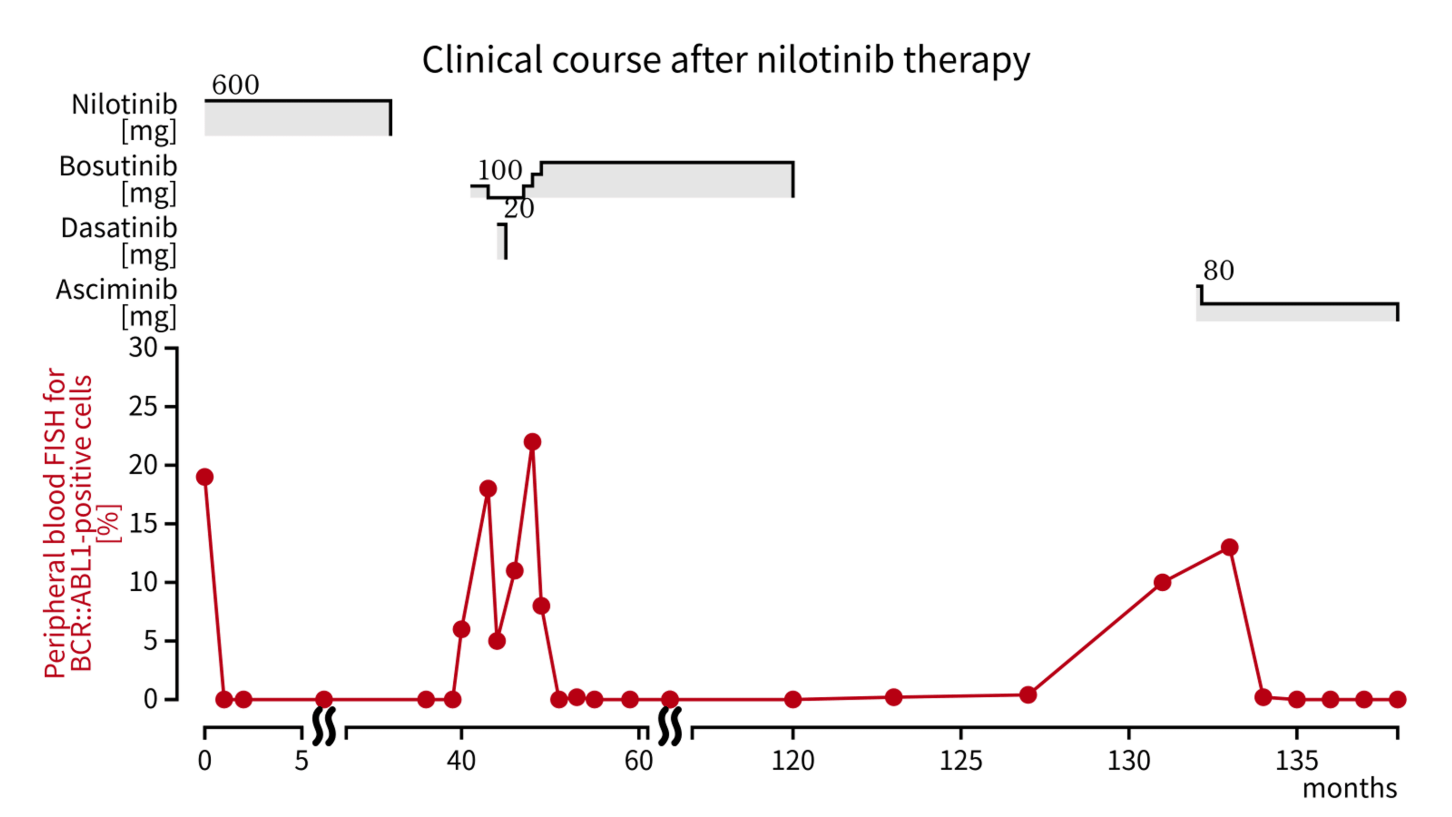
Clinical course of our case after nilotinib therapy The patient was treated with nilotinib for 32 months, but it was stopped due to cerebral infarction and atrial fibrillation. After loss of complete cytogenetic response (CCyR), bosutinib was introduced and two months later interrupted due to hepatotoxicity. Dasatinib was started but had to be discontinued within a month due to a transient ischemic attack. Then bosutinib was reintroduced and continued for 73 months until acute heart failure occurred. Eleven months after the discontinuation of bosutinib, CCyR was lost again and asciminib was initiated. Three months after starting asciminib, peripheral blood interphase fluorescence in situ hybridization showed 0% *BCR::ABL1*-positive cells and CCyR was achieved.

The patient remained untreated and was observed until September 2023 when he lost CCyR again. Asciminib 40 mg twice daily was initiated in October 2023. Blood tests at the start of asciminib treatment showed 10% *BCR::ABL1*-positive cells in peripheral blood FISH (Figure 1) testing and the bone marrow test showed 1.6% blasts, with only -Y as an additional chromosomal abnormality, which was the same as the initial diagnosis; no mutations, including T315I, were detected in the *BCR::ABL1* fusion gene. Qualitative RT-PCR testing confirmed micro *BCR::ABL1* (e19a2 transcript) positivity (Figure 2). Five days after starting asciminib at 40 mg twice daily, the dose was reduced to 20 mg twice daily due to thrombocytopenia. Three months later, peripheral blood FISH showed 0% *BCR::ABL1*-positive cells, indicating CCyR (Figure 3). The patient continued to maintain CCyR through April 2024. In May 2024, the patient experienced cerebral infarction; however, due to the preexisting severe stenosis of the internal carotid and basilar arteries before starting asciminib, the likelihood of a causal relationship between asciminib and the stroke was considered low, and asciminib treatment was continued.

## Discussion

To our knowledge, only two cases of CML patients with the e19a2 micro *BCR::ABL1* transcript successfully treated with asciminib have been reported (Table 2) [17,18]. One patient discontinued asciminib and maintained treatment-free remission (TFR) [18]. Both reported cases had the T315I mutation and the dosage of asciminib was 160 or 150 mg daily, which is higher than the recommended dosage in non-T315I mutation patients in the chronic phase. In contrast, our patient did not have the T315I mutation, and this is the first reported case in which asciminib was effective at a dose of 80 mg per day or less in a CML patient with the e19a2 micro *BCR::ABL1* transcript.

**Table 2:** Cases of CML patients with the e19a2 micro BCR::ABL1 transcript successfully treated with asciminib CCyR: complete cytogenetic response; DMR: deep molecular response; MR 5: 1 or less out of every 100,000 cells (0.001% or less) have the *BCR::ABL1* transcript

Case	Age/sex	Previous TKI therapy	Best response with asciminib	Study
1	60s/F	Imatinib	DMR(MR5)	Pagani et al., 2020 [17]
2	30s/M	Nilotinib, ponatinib	DMR(MR5)	Ernst et al., 2024 [18]
Present case	80s/M	Imatinib, nilotinib, bosutinib, dasatinib	CCyR	

Recent studies have suggested that, when compared to patients with the typical major *BCR::ABL1* transcript, patients with the e19a2 micro *BCR::ABL1* transcript have lower response rates to imatinib, reduced two-year event-free survival (EFS) and progression-free survival (PFS), and may respond better to second-generation TKIs [9,10]. Compared to the typical CML subtype, one reason for the poor response of patients with the e19a2 micro *BCR::ABL1* transcript is the higher likelihood of *BCR::ABL1* mutations arising after TKI initiation [5]. However, no *BCR::ABL1* mutations were detected in our case. In routine clinical practice, clinicians face some challenges when treating patients with the e19a2 transcript compared with those with the typical major *BCR::ABL1* transcript. First, diagnosis is difficult. Until recently, it was infeasible to measure e19a2 transcripts by RT-PCR in standard clinical practice in Japan. Second, there are treatment and adaptation challenges. The National Comprehensive Cancer Network (NCCN) and the European LeukemiaNet (ELN) guidelines recommend that the depth of response based on *BCR::ABL1 *international scale (IS) levels be used to determine the treatment strategy [11,12]. For example, changes in TKI treatment are considered if major molecular response (MMR; *BCR::ABL1 *IS≤0.1%) is not achieved, and decisions regarding TFR are based on the achievement of deep molecular response (DMR; *BCR::ABL1 *IS≤0.01% or 0.0032%). However, *BCR::ABL1 *IS levels can be measured only in patients with the major *BCR::ABL1* transcript. Monitoring MMR or DMR in patients with the e19a2 transcript is uncommon in normal clinical practice, complicating treatment decisions aimed at achieving MMR or TFR. Third, adverse event management can be difficult. The inability to accurately measure the depth of response makes it complicated to decide on dose reduction or treatment interruptions when adverse events occur.

In our case, it was challenging to monitor responses deeper than CCyR, and TKIs had to be discontinued because of cardiovascular events, resulting in cycles of FISH positivity (loss of CCyR) and resumption of TKIs. This difficulty in managing the side effects of TKIs prevented continuous TKI administration of sufficient intensity. Asciminib binds to the myristoyl pocket of ABL1 and induces an inactive conformation of the *BCR::ABL1* fusion protein and inhibits CML cell proliferation [19]. Although some reports have demonstrated the efficacy and safety of asciminib [13,15,19,20], these analyses were limited to patients with the major *BCR::ABL1* transcript, leaving the treatment effects on other subtypes unclear. Only two case reports have shown that asciminib is effective in patients with the e19a2 micro *BCR::ABL1* transcript [17,18]. These two cases had T315I mutation that is resistant to imatinib or second-generation TKIs, and our case was intolerant to conventional TKIs rather than resistant. Therefore, in this report, we cannot compare the efficacy of asciminib with conventional TKIs in patients with the e19a2 micro *BCR::ABL1* transcript, but the two previous cases and our case suggest that the ABL1 myristoyl pocket remains intact in the e19a2 *BCR::ABL1* transcript. Given that asciminib acts on the myristoyl pocket of ABL1, it could be hypothesized that asciminib would also be effective in patients with the minor *BCR::ABL1* or micro *BCR::ABL1* transcript, which has different *BCR* breakpoints from the major *BCR::ABL1* transcript. Recently, some cases have reported that rare *ABL1* breakpoint translocations, such as the e13a3 and e14a3 major *BCR::ABL1* transcripts, can lose their functional SH3 domains and are therefore resistant to asciminib, even if the myristoyl pocket is intact [21]. The breakpoint or mutation of the *ABL1* gene may have a more critical impact on resistance to asciminib than that of the *BCR* gene.

Vascular adverse events, including pulmonary hypertension and venous and arterial occlusive diseases, are well-recognized complications associated with second- and third-generation TKIs [22]. Although long-term follow-up data from the ASCEMBL study indicated that asciminib did not increase the risk of arterial occlusive events [23], further investigations are warranted to comprehensively evaluate the incidence of cardiovascular events with its extended use. Due to its minimal off-target effects, asciminib is expected to have lower cardiovascular toxicity than conventional ATP-competitive TKIs. Although our patient experienced cerebral infarction after starting asciminib treatment, severe stenosis of the internal carotid and basilar arteries was present at the time of asciminib initiation. It might be recommended that asciminib should be introduced before the long-term use of conventional TKIs, which carry the risk of causing irreversible arterial disease. This point applies to patients with the e19a2 transcript and to patients with other subtypes as well, but clinicians should consider cardiovascular events more carefully in patients with the e19a2 transcript. These patients are often resistant to imatinib and may require long-term treatment with second-generation TKIs.

This study has some limitations as well. One is that the response might be more accurately evaluated using quantitative RT-PCR for the e19a2 micro *BCR::ABL1* transcript; however, we were unable to measure it because quantitative RT-PCR for the e19a2 transcript is not commercially available in Japan. In addition, the follow-up period remains relatively short, and longer observation is needed to assess the durability of the treatment response.

## Conclusions

In conclusion, asciminib is effective for CML patients with the e19a2 micro *BCR::ABL1* transcript. Further accumulation of data on cases treated with asciminib for this subtype is necessary to draw more definitive conclusions. Additionally, future studies should focus on the long-term outcomes and safety profile of asciminib in this patient population.
